# Predictors of Perceived Need for and Prescribing of Digital Health Applications for Mental Disorders Among Psychotherapists in Germany: Cross-Sectional Survey Study

**DOI:** 10.2196/78597

**Published:** 2025-11-20

**Authors:** Esther Stalujanis, Deborah Engesser, Pascal Kemmerer, Lena Dotzauer, Sandra Salm, Sandy Scheibe, Karola Mergenthal, Karen Voigt, Susanne Singer

**Affiliations:** 1 Division of Epidemiology and Health Services Research Institute of Medical Biostatistics, Epidemiology and Informatics (IMBEI) University Medical Center of the Johannes Gutenberg University Mainz Mainz Germany; 2 Department of Consultation Psychiatry and Psychosomatic Medicine University Hospital of Zürich Zürich Switzerland; 3 Clinical Psychology and Psychotherapy – Methods and Approaches Department of Psychology University of Trier Trier Germany; 4 Institute of General Practice Goethe University Frankfurt Frankfurt am Main Germany; 5 Medical Psychology – Neuropsychology and Gender Studies and Center for Neuropsychological Diagnostics and Intervention (CeNDI) Faculty of Medicine and University Hospital Cologne University of Cologne Köln Germany; 6 Department of General Practice Dresden University of Technology Faculty of Medicine Carl Gustav Carus Dresden Germany; 7 Department of Quality of Life in Oncology Comprehensive Cancer Center Mecklenburg-Vorpommern (CCC-MV) University Medical Centre Rostock Rostock Germany

**Keywords:** cross-sectional study, digital health applications, health services research, mental health, mobile health, mHealth, perceived need, prescribing

## Abstract

**Background:**

Since 2020, psychotherapists in Germany have been allowed to prescribe digital health applications for mental disorders (DHA-MDs). DHA-MDs were supposed to foster digitalization and improve access to mental health services. However, prescriptions of DHA-MDs are less frequent than expected.

**Objective:**

We aimed to investigate which characteristics of psychotherapists predicted perceived need for and prescribing of DHA-MDs among German psychotherapists.

**Methods:**

This study was a cross-sectional survey conducted in Germany. We contacted psychotherapists between January 2024 and April 2024 via professional associations, training institutes, or the social media platform X (formerly known as Twitter). We sent questionnaires to 1000 psychotherapy practices randomly selected from the registry of the National Association of Statutory Health Insurance Physicians. We assessed the characteristics of the psychotherapists, asked them to rate the perceived need for DHA-MDs on an 11-point scale, and documented their prescribing behavior. Linear and logistic regression analyses were performed to predict perceived need and prescribing, respectively.

**Results:**

A total of 321 psychotherapists participated. Perceived need was estimated as low (mean 2.1, SD 2.1). Among the 271 (84.4%) psychotherapists with valid data, 83 (30.6%) prescribed a DHA-MD to at least 1 patient in a regular quarter. As compared to a behavioral approach, a psychodynamic (b=–1.63; *P*<.001) or systemic psychotherapy approach (b=–1.48; *P*=.005) and higher age (b=–0.03; *P*=.007) negatively predicted the perceived need, while being a physician (vs psychologist; b=0.73; *P*=.02) and a psychotherapeutic and psychopharmacological treatment focus (vs psychotherapeutic alone; b=1.31; *P*=.04) positively predicted the perceived need. For prescribing DHA-MDs, odds were lower for psychotherapists with a psychodynamic, systemic, or other approach (vs behavioral approach; odds ratio [OR] 0.30, 95% CI 0.14-0.64). The odds were higher for psychotherapists with more than a half service mandate (vs less than or equal to a half service mandate; OR 2.99, 95% CI 1.50-5.97), working in a group practice or medical care center (vs single practice; OR 2.39, 95% CI 1.04-5.53), and being located in a rural community or small town (OR 2.74, 95% CI 1.27-5.94) or a medium-sized town (OR 2.67, 95% CI 1.21-5.92) compared to a large city.

**Conclusions:**

Professional group, psychotherapy approach, age, the size of service mandate, treatment focus, practice type, and community size predicted perceived need for and prescribing of DHA-MDs among psychotherapists. Our findings may inform stakeholders and decision-makers in health care and politics about where DHA-MDs have already been implemented in the landscape of mental health services.

## Introduction

Mental disorders constitute a substantial global burden of disease and contribute to high costs for the health care system [1]. Due to the limited availability of face-to-face psychotherapy, a large percentage of people with mental disorders have limited access to psychotherapy and are confronted with long waiting times [2-4].

Because the World Health Organization defined the development of mobile technologies and ITs as one of their research priorities to increase access to evidence-based care [5], the fields of eHealth and mobile health (mHealth) evolved rapidly [6]. Several national initiatives emerged in parallel, such as, Belgium’s mHealth validation pyramid (2018), which aimed to standardize app assessment [7]; the US Food and Drug Administration’s Digital Health Innovation Action Plan (2017), which laid the foundation for digital health precertification [8]; and the United Kingdom’s National Health Service Apps Library (2017), later extended through National Health Service Digital Pathways and Digital Technology Assessment Criteria evaluation frameworks [9]. Building on these developments, in 2019, Germany became the first country to establish a comprehensive legal framework for prescribing and reimbursing digital health applications (DHAs; German: Digitale Gesundheitsanwendungen [DiGA]) through the Digital Healthcare Act and the DiGA fast track [10]. This act provided the legal basis for the prescription of DHAs by physicians and psychotherapists as well as their reimbursement of DHAs via the statutory health insurance, aiming to foster digitalization and improve access to health care, including psychotherapy [11]. DHAs need to fulfill usability aspects and data security and safety requirements and provide proof of a *positive care effect*, which can be a medical benefit or a contribution to structural and process improvement, to be permanently approved by the Federal Institute for Drugs and Medical Devices (German: Bundesinstitut für Arzneimittel und Medizinprodukte) [12]. Among all DHAs temporarily or permanently approved, those addressing mental disorders (DHAs for mental disorders [DHA-MDs]; German: Digitale Gesundheitsanwendungen für psychische Erkrankungen [PsyDiGA]) reached a cumulative market share of approximately 31%, based on the summed market shares of all DHA-MDs, between October 1, 2020, and June 30, 2023 [12,13]. Notably, DHA-MDs are mostly conceptualized as stand-alone digital tools to be used directly by patients. This feature distinguishes them from other digital health platforms or apps that may support face-to-face psychotherapy.

DHA-MDs have been designed to help bridge waiting times to ambulatory face-to-face psychotherapy. However, prescription rates are low so far. One of the largest health insurance companies in Germany, the Techniker Krankenkasse, reported having issued about 86,000 DHA activation codes to 69,000 out of 11.1 million insured people, from October 2020 until the end of June 2023, which corresponds to a proportion of 0.6%. The share for DHA-MDs was 26.500 activation codes issued in the same period [13]. A total of 15% of the DHA prescriptions are being issued by psychiatrists and psychotherapists, of whom most are still quite reserved in prescribing DHAs or DHA-MDs [13]. Reported reasons include concerns regarding data security, a lack of evidence for the efficacy of DHA-MDs, technical barriers, and a lack of knowledge among health care professionals [14-18]. Concerns about the robustness of evidence on the efficacy and effectiveness of DHA-MDs are consistent with recent systematic reviews of approval studies. These reviews reported medium to large effect sizes, suggesting that DHA-MDs can be beneficial in reducing mental health disorder symptoms. At the same time, they highlighted important methodological limitations, such as small sample sizes, short intervention and follow-up periods, and a generally high risk of bias. Thus, while findings on efficacy and effectiveness are promising, the robustness of this evidence remains limited, and further high-quality, large-scale trials are needed. Consequently, skepticism among psychiatrists and psychotherapists may be partly justified, and has been reflected in ongoing discussions about the approval process of DHA-MDs, which has been criticized for not sufficiently ensuring study quality [19-21].

To date, there is little research on the perceived need for DHA-MDs and prescribing among psychotherapists. Previous studies focused mainly on physicians or other health care professionals [15-17,22-24]. Furthermore, only a few studies have investigated the association between characteristics of health care professionals and their perceived need for DHA-MDs as well as their prescribing behavior. Brecher et al [25] reported more positive attitudes and greater willingness to prescribe DHA among younger family physicians, whereas Staeck et al [26] did not find an association between acceptance of e–mental health and age in a sample of psychotherapists in training. In a Portuguese study [27], psychologists viewed digital mental health apps more favorably compared to psychiatrists. Other studies found that acceptance differed between theoretical orientations, with more skepticism among psychotherapists with a psychodynamic orientation as compared to a behavioral orientation [26,28-30]. The studies mentioned differed largely in professional groups and countries included, and outcomes were mainly attitudes or acceptance of digital mental health applications.

In our study, we investigated the association between characteristics of psychotherapists as well as their practices and perceived need for DHA-MDs and prescribing of DHA-MDs.

## Methods

### Study Design

We conducted a cross-sectional survey in Germany between January 2024 and April 2024 as part of the study Evaluation of Digital Health Applications for Mental Disorders (Digitale Gesundheitsanwendungen für psychische Erkrankungen auf dem Prüfstand [DiGAPS]), funded by the Federal Joint Committee (01VSF22022).

### Ethical Considerations

The ethics committee of the State Medical Association of Rhineland-Palatinate *(*Landesärztekammer Rheinland-Pfalz) approved the study protocol of the DiGAPS study (2023-17268). All participants provided informed consent for study participation. Data of participants who participated only in this survey were assessed anonymously. Data of psychotherapists who also participated in other parts of the DiGAPS project were assessed in a pseudonymized form. Data were not linked between the different parts of the study. Participants did not receive any compensation for participation.

### Data Collection and Procedures

In January 2024, we sent a link to the online version of the questionnaire to the mailing lists of professional associations of psychotherapists and training institutes of psychotherapy. We also provided the link on the social media platform X (formerly known as Twitter; X Corp) via the account of the principal investigator (S Singer). There were no inclusion or exclusion criteria for the online survey. In addition, in February 2024, we sent the questionnaires by mail to a random sample of 1000 psychotherapy practices located in Germany, which we had drawn out of the official registry of the National Association of Statutory Health Insurance Physicians, stratified according to the proportion of inhabitants of Germany, federal state, and community size. In the information sheet provided to participants, we explained that the questions referred exclusively to the German DiGA concept and, more specifically, to DiGAs for mental disorders (DHA-MD).

### Assessment of Psychotherapists’ and Practices’ Characteristics

We developed a questionnaire in which the following characteristics of psychotherapists and their practices were assessed: gender (man, woman, or nonbinary), age, years of professional experience, professional group (physician [psychiatry or psychosomatic medicine], psychologist, or other), age focus (adults, children and adolescents, or both), psychotherapy approach (low-frequency psychodynamic psychotherapy, psychoanalytic psychotherapy, behavioral therapy, or systemic therapy), treatment focus (psychotherapeutic, psychopharmacological, or both), practice type (single practice, group practice, medical care center [German: Medizinisches Versorgungszentrum], or joint practice), size of service mandate (quarter, half, three-quarter, whole, or none), and community size (large city [>100,000 inhabitants], medium-sized town [20,000-100,000 inhabitants], small town [5000-20,000 inhabitants], rural community [<5000 inhabitants], or unknown). In this study, we used the term psychotherapists to refer to both physicians (psychiatry and psychosomatic medicine) and psychologists.

### Assessment of Perceived Need for DHA-MDs and Prescription Behavior

The perceived need for DHA-MDs was assessed with the item “What is the need for DHA-MD among your patients?” (German: “Wie hoch schätzen Sie den Bedarf an PsyDiGA bei Ihren Patient:innen ein?”) on an 11-point scale from 0 (very low) to 10 (very high). The 11-point format was chosen to allow for sufficient sensitivity and differentiation and is consistent with numerical rating scales widely used in health research [31,32] to capture subjective perceptions, such as pain or fatigue. The item was developed within the DiGAPS project and refined after pilot testing to enhance clarity and comprehensibility. We assessed the number of prescriptions by asking respondents how many patients they typically prescribe a DHA-MD (German: PsyDiGA) to in a regular quarter (German: “Wie vielen Patient:innen verschreiben Sie in einem gewöhnlichen Quartal eine PsyDiGA?”). Respondents were instructed to access their practice documentation (eg, electronic practice management system) or provide estimates if documentation was not available.

### Statistical Analyses

We determined the a priori sample size based on the rule of thumb of requiring 10 to 20 observations per predictor level [33]. We planned to include 13 predictor levels, which resulted in a target sample size of 260.

We conducted the statistical analyses with Stata (version 15.1; StataCorp) [34]. For categorical variables, we calculated absolute and relative frequencies. For continuous variables, we calculated means, medians, and SDs and, where appropriate, ranges. We dichotomized the number of prescriptions (0 [no DHA-MD prescribed] vs 1 [at least 1 DHA-MD prescribed in a regular quarter]). We grouped psychotherapy approaches as follows: “psychodynamic” if a therapist stated either low-frequency psychodynamic psychotherapy, psychoanalytic psychotherapy, or both and “systemic or other” if a therapist stated systemic and another approach or another combination. Size of service mandate was summarized as follows: “greater than half” for three-quarters or whole, “less than or equal to half” for half and quarter, and “none” in case of no service mandate (eg, private practice).

Predictors of perceived need were investigated using linear regression analyses. We conducted separate crude linear regression models with the following variables as predictors: age, years of professional experience, gender, professional group, age focus, psychotherapy approach, size of service mandate, treatment focus, practice type, and community size. Interaction terms were tested for gender×professional type, age×therapy approach, and age×gender and included in the full model if they were statistically significant. We checked for multicollinearity by inspecting the variance inflation factor. In addition, we examined bivariate correlations between conceptually overlapping variables (eg, age and years of professional experience). Variables showing problematic collinearity with other predictors were not entered into the full models. Model fit was tested using maximum likelihood ratio tests. We inspected residual plots to check that the assumptions for linear regression were not violated. We reported the number of participants per predictor category, regression coefficients as unstandardized b, representing the change in the dependent variable per 1-unit increase of the predictor (ie, per year for age and per category relative to the reference for categorical predictors), 95% CIs, and *P* values as well as the adjusted *R*^2^.

Predictors of prescribing DHA-MDs (yes vs no) were explored with binary logistic regression analyses. We used the same predictor categories as described earlier for the linear regression models. We examined the number of prescribing events per predictor category, and categories with fewer than 10 events were collapsed with conceptually similar groups to ensure model stability (refer to Multimedia Appendix 1 for details on recoding). Furthermore, we excluded age focus from the full model because current DHA-MDs are only eligible for adult patients. Treatment focus was also excluded from the full model to avoid overfitting and due to its conceptual overlap with the professional group. However, for reasons of transparency, treatment focus was retained in the crude models. Interaction terms were tested for gender×professional type, age×therapy approach, and age×gender and included in the full model if they were statistically significant. Model fit was tested using maximum likelihood ratio tests. We checked for multicollinearity by comparing the pseudo *R*^2^ of the models with and without highly correlating variables and included only one of the variables if that did not significantly impair model fit. We reported events per predictor for each category included in the full model, events per variable (EPV) for the model, odds ratios (ORs), 95% CIs, and *P* values.

We restricted each analysis to participants with complete data (refer to the Results section). Statistical significance was evaluated using 2-sided tests with an α level of .05.

## Results

### Characteristics of the Sample

**Figure 1:**
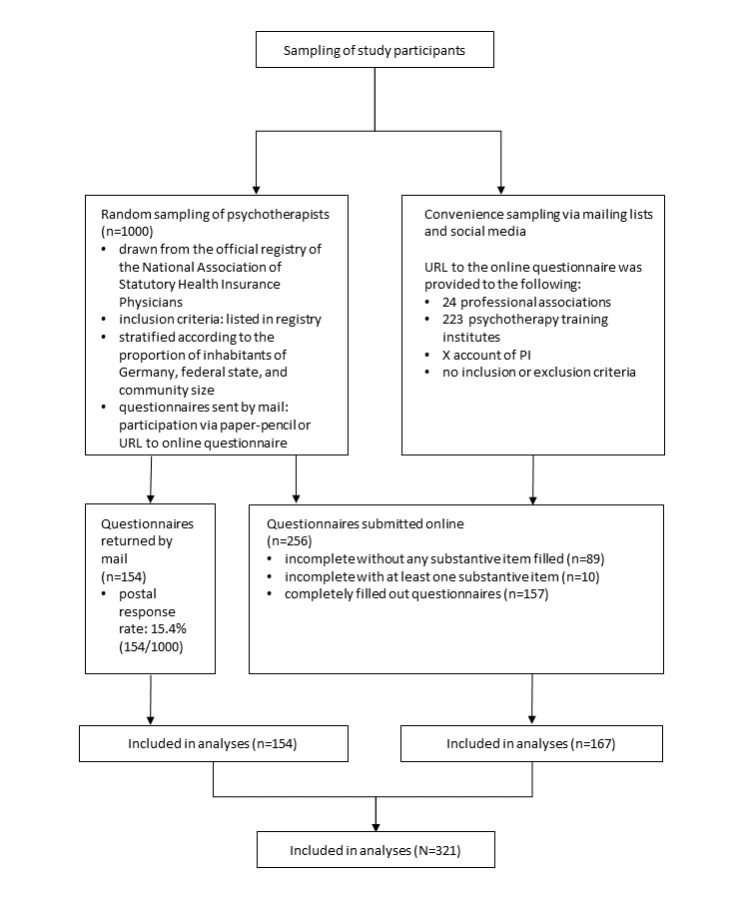
Flowchart of the study participants (N=321). PI: principal investigator.

The flow of study participants is depicted in Figure 1. The characteristics of the 321 study participants are described in Table 1.

**Table 1:** Characteristics of the study sample (N=321)^a^.

Categorical variables		Values
**Gender (n=319), n (%)**		
	Woman	229 (71.8)
	Man	89 (27.9)
	Nonbinary	1 (0.3)
**Professional group (n=320), n (%)**		
	Psychologist	237 (74.1)
	Physician (psychiatry or psychosomatic medicine)	75 (23.4)
	Other^b^	8 (2.5)
**Age focus (n=310), n (%)**		
	Adults	278 (89.7)
	Children and adolescents	11 (3.5)
	Children, adolescents, and adults	21 (6.8)
**Psychotherapy approach (n=306), n (%)**		
	Psychodynamic	170 (55.6)
	Behavioral	119 (38.9)
	Systemic or other	17 (5.6)
**Size of service mandate (n=316), n (%)**		
	Less than or equal to half	194 (61.4)
	Greater than half	102 (32.3)
	None	20 (6.3)
**Treatment focus (n=319), n (%)**		
	Psychotherapeutic	294 (92.2)
	Psychopharmacological	9 (2.8)
	Both	16 (5)
**Practice type (n=317), n (%)**		
	Single practice	196 (61.8)
	Group practice	42 (13.2)
	Medical care center	11 (3.5)
	Joint practice	68 (21.5)
**Community size (n=316), n (%)**		
	Rural community (<5000 inhabitants)	27 (8.5)
	Small town (5000-20,000 inhabitants)	64 (20.3)
	Medium-sized town (>20,000-100,000 inhabitants)	71 (22.5)
	Large city (>100,000 inhabitants)	154 (48.7)
**Numeric variables (y), mean (SD)**		
	Age (n=318)	52.3 (13.0)
	Professional experience (n=319)	21.0 (12.0)

^a^Due to missing data, the sample population varies throughout categories.

^b^A few participants reported other professional backgrounds, including child and adolescent psychotherapists (Kinder- und Jugendlichen-Psychotherapeut:innen; for whom different training requirements applied), which is consistent with historical training pathways and transition regulations in Germany before the Psychotherapist Act of 1998.

### Perceived Need and Prescribing Behavior

**Figure 2:**
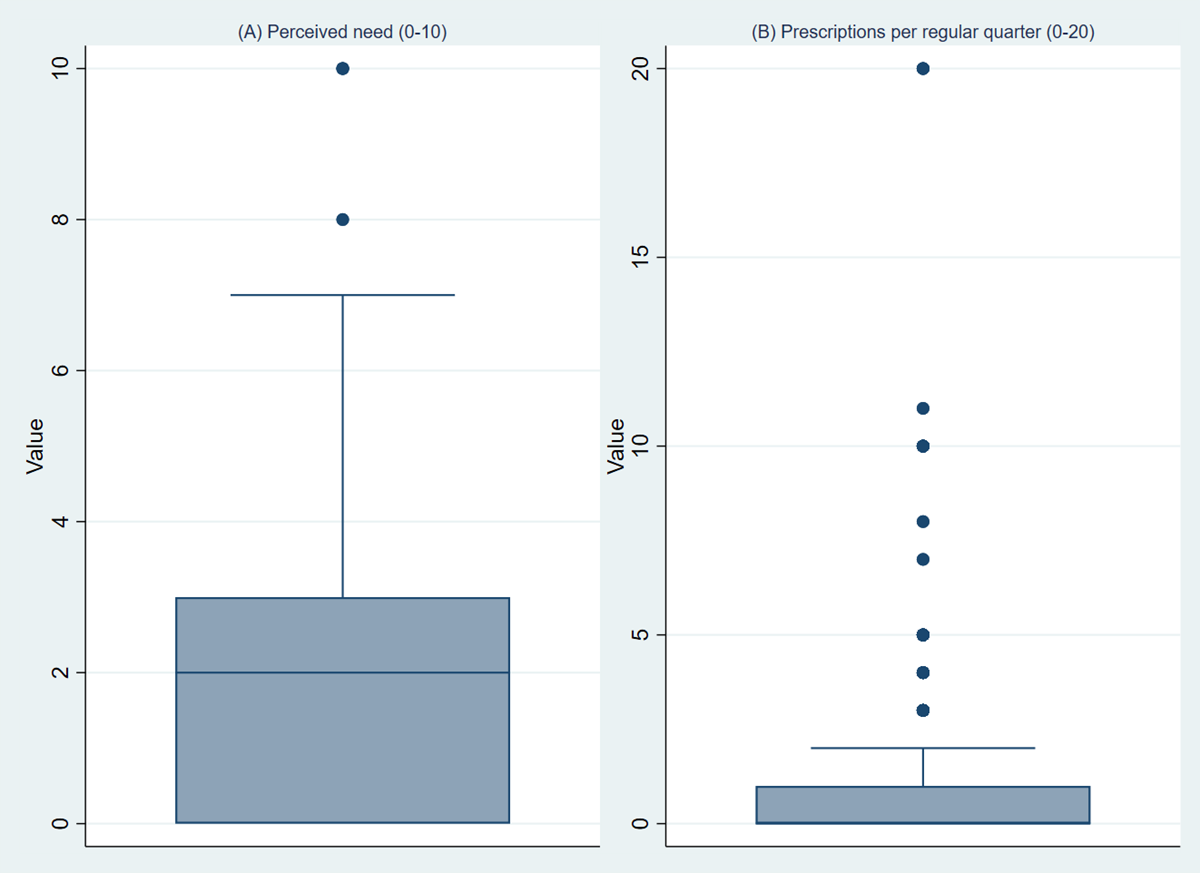
Boxplots of (A) perceived need for digital health applications for mental disorders (0-10 scale) and (B) number of digital health applications for mental disorder prescriptions per quarter. For perceived need, the full range of the response scale (0-10) was used. According to boxplot rules, scale end points are displayed as outliers, although they represent valid response options.

A total of 308 (95.9%) of the 321 participants reported a perceived need for DHA-MDs. The average perceived need was 2.09 (SD 2.13; median 2, IQR 3) on a scale from 0 (very low) to 10 (very high). As illustrated in Figure 2, the distribution of perceived need covered the full range of the scale (0-10), with most responses in the lower range.

The number of patients to whom psychotherapists typically prescribed a DHA-MD in a regular quarter ranged from 0 to 20 (mean 1.1, SD 2.65; median 0, IQR 1). The distribution of prescription counts (Figure 2) was also highly skewed, with most (212/271, 78.2%) respondents reporting no or only 1 prescription per regular quarter and only a few (3/271, 0.1%) respondents reporting higher numbers (>10 per quarter). For the binary outcome used in logistic regression analyses, among the 271 respondents with valid data, we found that 83 (30.6%) psychotherapists prescribed at least 1 DHA-MD per quarter. Further details on perceived need and prescribing behavior are reported in a separate publication by our group (Engesser et al, unpublished data, November 2025).

### Psychotherapists’ Characteristics as Predictors of Perceived Need

As age and years of professional experience correlated highly (*r*=0.87; *P*<.001), we did not enter years of professional experience in the full models. Exploratory analyses of interaction terms (gender×age, gender×professional group, and age×psychotherapy approach) did not yield evidence of effect modification and were therefore not included in the full model.

In the full linear regression model adjusting for each of the other variables (Table 2), 281 participants were included, encompassing 26 predictor levels, corresponding to approximately 10.8 observations per predictor level. The associations mentioned subsequently were statistically significant. Practicing as a physician (psychiatry or psychosomatic medicine) was associated with higher perceived need compared to practicing as psychologist (b=0.73, 95% CI 0.12-1.35; *P*=.02). A psychotherapeutic and psychopharmacological (both) treatment focus was associated with higher perceived need compared to a psychotherapeutic treatment focus (b=1.31, 95% CI 0.04-2.59; *P*=.04). Engaging in a psychodynamic (b=–1.63, 95% CI –2.18 to –1.09; *P*<.001) or systemic or other approach was associated with lower perceived need as compared to a behavioral approach (b=–1.48, 95% CI –2.53 to –0.44; *P*=.005). Higher age (per 1-year increase; b=–0.03, 95% CI –0.05 to –0.01; *P*=.007) negatively predicted the perceived need. Adjusted *R*^2^ for the model was 0.23. Results of the crude models are presented in Multimedia Appendix 2.

**Table 2:** Full linear regression model for psychotherapists’ characteristics as predictors of perceived need for digital mental health applications (n=281).

Predictor variable			Psychotherapists, n (%)		b (95% CI)		*P* value
**Gender^a^**							
	Woman (reference)	201 (71.5)		—^b^		—	
	Man	80 (28.5)		–0.11 (–0.62 to 0.41)		.68	
**Professional group**							
	Psychologist (reference)	211 (75.1)		—		—	
	Physicians (psychiatry or psychosomatic medicine)	*63 (22.4)* ^c^		*0.73* (0.12 to 1.35)		*.02*	
	Other	7 (2.5)		–0.76 (–2.99 to 1.48)		.51	
**Age focus**							
	Adults (reference)	253 (90)		—		—	
	Children and adolescents	9 (3.2)		0.73 (–1.29 to 2.74)		.48	
	Both	19 (6.8)		0.72 (–0.16 to 1.59)		.11	
**Psychotherapy approach**							
	Psychodynamic	158 (56.2)		–*1.63* (–2.18 to –1.09)		**<** *.001*	
	Behavioral (reference)	107 (38.1)		—		—	
	Systemic or other	16 (5.7)		–*1.48* (–2.53 to –0.44)		*.005*	
**Size of service mandate**							
	Less than or equal to half (reference)	176 (62.6)		—		—	
	Greater than half	91 (32.4)		0.12 (–0.39 to 0.63)		.64	
	None	14 (5)		0.41 (–0.66 to 1.49)		.45	
**Treatment focus**							
	Psychotherapeutic (reference)	263 (93.6)		—		—	
	Psychopharmacological	6 (2.1)		0.87 (–0.72 to 2.46)		.28	
	Both	12 (4.3)		*1.31* (0.04 to 2.59)		*.04*	
**Practice type**							
	Single practice (reference)	177 (63)		—		—	
	Group practice	38 (13.5)		0.35 (–0.34 to 1.05)		.32	
	Medical care center	10 (3.6)		0.94 (–0.32 to 2.21)		.14	
	Joint practice	56 (19.9)		0.52 (–0.06 to 1.10)		.08	
**Community size**							
	Rural community (<5000 inhabitants)	26 (9.3)		0.31 (–0.51 to 1.14)		.46	
	Small town (5000-20,000 inhabitants)	58 (20.6)		0.31 (–0.33 to 0.95)		.34	
	Medium-sized town (>20,000-100,000 inhabitants)	64 (22.8)		0.49 (–0.09 to 1.08)		.10	
	Large city (>100,000 inhabitants; reference)	133 (47.3)		—		—	
Age (y)			281 (100)		–*0.03* (–0.05 to –0.01)		*.007*

^a^In the variable “gender,” for “nonbinary,” there was only 1 case; therefore, we set it to missing and skipped the category but kept the person in the dataset.

^b^Not available.

^c^Italicized values indicate statistical significance.

### Psychotherapists’ Characteristics as Predictors of Prescribing Behavior

Exploratory analyses of interaction terms (gender×age, gender×professional group, and age×psychotherapy approach) did not yield evidence of effect modification and were therefore not included in the full model.

The full logistic regression model (Table 3) was based on 230 participants, yielding an EPV ratio of 8.33. Categories with too few events were merged; the details are provided in Multimedia Appendix 1. Odds for prescribing DHA-MDs were lower for psychotherapists with a psychodynamic, systemic, or other approach as compared to a behavioral approach (OR 0.30, 95% CI 0.14-0.64). Odds for prescribing DHA-MDs were higher for psychotherapists with more than a half service mandate compared to less than or equal to half a service mandate (OR 2.99, 95% CI 1.50-5.97) if they worked in a group practice or medical care center (OR 2.39, 95% CI 1.04-5.53) compared to a single practice and if they were located in a rural community or small town (OR 2.74, 95 CI 1.27-5.94) or a medium-sized town (OR 2.67, 95% CI 1.21-5.92) compared to a large city. Results of the crude models are presented in Multimedia Appendix 3.

**Table 3:** Full logistic regression model for psychotherapists’ characteristics as predictors of prescribing a digital mental health application (n=230).

Predictor variable		Events per predictor (prescribing: 1=yes)	OR^a^ (95% CI)	*P* value
**Gender^b^**				
	Female (reference)	65	—^c^	—
	Male	17	0.61 (0.29-1.25)	.18
**Professional group**				
	Psychologist (reference)	60	—	—
	Physician (psychiatry or psychosomatic medicine)	23	2.21 (0.99-4.96)	.05
**Psychotherapy approach**				
	Psychodynamic, systemic, or other	31	*0.30*^d^ (0.14-0.64)	**<** *.002*
	Behavioral (reference)	46	—	—
**Size of service mandate**				
	Less than or equal to half (reference)	45	—	—
	More than half	38	*2.99* (1.50-5.97)	**<** *.002*
**Practice type**				
	Single practice (reference)	46	—	—
	Group practice or medical care center	23	*2.39* (1.04-5.53)	*.04*
	Joint practice	13	1.52 (0.67-3.48)	.32
**Community size**				
	Rural community or small town (≤20,000 inhabitants)	30	*2.74* (1.27-5.94)	*.01*
	Medium-sized town (>20,000-100,000 inhabitants)	25	*2.67* (1.21-5.92)	*.02*
	Large city (>100,000 inhabitants; reference)	27	—	—
Age		83	0.98 (0.95-1.01)	.20

^a^OR: odds ratio.

^b^In the variable “gender,” for “nonbinary,” there was only 1 case; therefore, we set it to missing and skipped the category but kept the person in the dataset. Events per predictor refer to cases in which participants stated that they prescribed at least 1 digital health application for mental disorders in a regular quarter.

^c^Not available.

^d^Italicized values indicate statistical significance.

## Discussion

We investigated which characteristics of psychotherapists predict their perceived need for and their prescribing of DHA-MDs. In our study, psychotherapists estimated the need for DHA-MDs as low, which is in line with a previous study [35] conducted with psychotherapists in training, where acceptance of e–mental health services was found to be lowest for unguided programs, comparable to DHA-MDs, conceptualized as stand-alone tools. The frequency of DHA-MD prescriptions was low, too. Only approximately 30.6% (83/271) of the psychotherapists usually prescribed DHA-MD, which is in line with previous studies [16,17], indicating that DHA-MDs are not prescribed on a broad scale yet.

The perceived need was higher among physicians than psychologists. In addition, odds of prescribing DHA-MDs were double for physicians compared to psychologists; however, this was not significant (*P*=.05). This is in contrast with a Portuguese study by Nogueira-Leite et al [27], in which psychologists were more in favor of DHA-MDs as compared to psychiatrists. However, the perceived need in our study was assessed considering the participants’ patients. Assuming that, on average, physicians treat more patients in a regular quarter compared to psychologists, they may not have enough capacity to offer adequate psychotherapeutic treatment to every patient and may welcome the opportunity to offer DHA-MDs, which may be used to supplement the regular psychiatric treatment or bridge waiting times. Our survey included an item asking whether participants would potentially prescribe DHA-MDs, particularly to bridge the waiting time for psychotherapy. However, these results are reported in a separate publication focusing on the application areas of DHA-MDs. To the best of our knowledge, no peer-reviewed studies have yet examined the use of DHA-MDs specifically in waiting-list contexts. However, evidence on digital interventions during psychotherapy waiting periods suggests that this option can be feasible and beneficial [36-38], indicating a promising future research direction for DHA-MDs. Perceived need and odds in the crude models for prescribing DHA-MDs were higher in physicians who focused on both psychotherapeutic and psychopharmacological treatments compared to physicians who focused on psychotherapeutic treatment alone. DHA-MDs may be supplemental to psychotherapy and comparable to psychopharmacological medication. A higher perceived need for and prescribing of DHA-MDs may be more obvious to physicians, who are used to prescribing additional components to psychotherapy. Furthermore, physicians may identify less strongly with their psychotherapeutic role than psychotherapists and thus be more willing to delegate this area of expertise. However, these interpretations are speculative and need to be further investigated in future studies.

Higher age predicted lower perceived need for prescribing DHA-MDs. Previous results regarding age as a predictor of acceptance and attitudes toward mHealth provided mixed findings. Brecher et al [25] reported more positive attitudes and higher willingness to prescribe DHAs in younger family physicians. In the latter-mentioned study, higher age was negatively associated with digital affinity [25], which may act as a confounding variable in the relationship between age and the perceived need for DHA-MDs. As we did not assess digital affinity in this analysis, we were unable to account for this factor. Therefore, future studies should include measures of digital affinity as well as other established predictors of digital health use, such as previous training, general attitudes toward technology, or practice infrastructure [28,39,40], to disentangle these mechanisms more clearly. Nevertheless, few studies have concluded that the age of psychotherapists or psychotherapists in training was not associated with attitudes concerning internet interventions [28] or acceptance of e–mental health services [26]. It should also be noted that such factors are being investigated in other parts of the DiGAPS project, including quantitative analyses based on data from general practitioners and qualitative interviews with psychotherapists and patients, published in separate papers.

The findings regarding gender were ambiguous. In the crude models, the male gender was associated with lower perceived need and lower odds for prescribing DHA-MDs, in line with previous studies [17,24]. We cannot confirm these findings in the adjusted models, in which gender did not predict acceptance or attitudes regarding mHealth, in line with other studies [26,28]. We initially hypothesized that age and gender as well as age and professional group might interact with each other. However, we were unable to confirm this.

The odds for prescribing DHA-MDs were higher for psychotherapists with more than half a service mandate. Those psychotherapists were obliged by statutory health insurance to offer a relatively high amount of psychotherapy sessions as well as consultation hours, which may bring them to a personal respectively emotional capacity limit. In this case, DHA-MDs may provide a greater number of patients with a low-threshold tool, particularly when regular psychotherapy cannot be offered (yet). Alternatively, higher service mandates may also be associated with greater exposure to professional training opportunities, more communication from professional associations of DHA-MD manufacturers, and better access to test accounts or implementation support, which could enhance familiarity with DHA-MDs and facilitate their use in practice. However, those explanations are tentative and need to be further explored.

In this regard, the odds of prescribing DHA-MDs are higher in group practices and medical care centers as compared to single practices. A potential explanation may be that in group practices or medical care centers, psychotherapists have more opportunities to share experience and information among peers and may be better informed about DHA-MDs, particularly when they have already gained positive attitudes toward eHealth [41]. A lack of knowledge and experience with DHA-MDs has been described as one of the main barriers to the use of DHAs [17,42,43].

A smaller community, as compared to a large city, was positively associated with perceived need (only in the crude models) and higher odds of prescribing DHA-MDs. This is in contrast to a study by Brecher et al [25] that reported that family physicians located in a larger city were better informed about DHAs and advised them more often to their patients compared to their colleagues from smaller towns. However, the capacities of psychotherapy are more limited in smaller towns and rural areas as compared to bigger cities [44], which may lead to a higher probability of prescribing DHA-MDs instead of offering no psychotherapeutic treatment when face-to-face psychotherapy is not available.

Another important predictor of the perceived need for and prescribing behavior of DHA-MDs was the approach or theoretical orientation of the psychotherapists. We found that psychodynamic and systemic psychotherapists estimated a lesser need for DHA-MDs compared to behavioral therapists. Furthermore, the odds for prescribing DHA-MDs were 70% lower for psychodynamic, systemic, or other psychotherapists compared to behavioral therapists. This finding has been consistently reported in previous studies beyond several mHealth applications and settings [26,28-30,45]. One reason for this finding might be that the currently listed DHA-MDs mainly use a cognitive behavioral approach. There is no DHA-MD based on a specifically psychodynamic or systemic therapy approach. This constitutes a large gap in mental health research and services. There are contemporary manualized and evidence-based psychotherapy methods based on psychodynamic conceptualizations, such as, for instance, mentalization-based treatment [46] for the treatment of borderline personality disorder and other mental disorders, where selected elements might be “translated” in digital mental health applications but have not yet been. Furthermore, there is evidence for the efficacy of internet interventions based on psychodynamic psychotherapy approaches [47-51]. However, previous studies reported more skepticism and less confidence among psychodynamic psychotherapists toward internet interventions as compared to psychotherapists of other theoretical orientations [28], which might be explained by underlying theoretical constructs and assumed mechanisms of action in psychodynamic psychotherapy, such as processing unconscious conflicts and working through transference (ie, the unconscious projection of internalized object representations onto the therapist). The reserved attitude toward digital mental health of psychodynamic psychotherapists runs through practice and research, which bears potential risks for psychodynamic psychotherapy as a whole. First, it may be beneficial from a content-related aspect to complement digital mental health applications with important transtheoretical mechanisms of action based on psychodynamic conceptualizations, such as mentalizing [52] or defense mechanisms [53]. Second, it is important for psychodynamic therapy and its implementation in mental health services not to lose track of future developments and technological progress in research and clinical practice. Although this field is still new, there are promising and innovative approaches, for instance, artificial intelligence in the form of large language models with elements of psychodynamic psychotherapy, such as creating psychodynamic case formulations based on ChatGPT [54]. Furthermore, a member of our group (ES) developed a prototype of an artificial intelligence assistant for the training of future psychotherapists in a mental health hackathon, which intends to detect ruptures in the therapeutic relationship and provide recommendations for repair [55]. These approaches indicate that, although difficult, it may be possible to translate psychodynamic conceptualizations into DHA-MDs.

A strength of our study is that it is the first comprehensive survey conducted specifically among psychotherapists in Germany, gathering data on perceived need for and prescribing of DHA-MDs, whereas previous studies included mixed samples, did not include actual prescribers of DHA-MDs, focused on acceptance as an outcome, or did not focus particularly on DHA-MDs [15-17,24,26-30]. Furthermore, our sample of psychotherapists is representative in terms of age [56] and professional group. However, it includes a slightly greater number of psychotherapists with a psychodynamic psychotherapy approach and fewer with a behavioral psychotherapy approach as compared to recent billing data [57].

Our study also has some limitations. First, our results may be affected by response bias. While we know which questionnaires were returned by mail and which were submitted online, we could not distinguish the online responses submitted by respondents from the randomly drawn registry sample from those recruited via mailing lists and social media. Psychotherapists who responded via professional networks or social media may differ systematically from those who responded via postal invitation regarding sociodemographic or practice characteristics. This may have introduced a selection bias with potential impact on the reported estimates. However, a stratified analysis was not feasible due to the limited traceability of the recruitment channel among online respondents. Furthermore, psychotherapists with a more positive attitude may have been more likely to participate, which may have led to an overreporting of perceived need and prescribing, as indicated by previous data from statutory health insurers [13]. It could also be the other way round, wherein an overproportioned ratio of psychotherapists with a more skeptical attitude may have participated, which may have potentially led to an underreporting of perceived need and prescribing. However, our findings regarding perceived need and prescribing behavior are in line with previous findings and thus seem to be at least comparable [16,17,35]. Second, because participation was largely anonymous and no personal identifiers were collected, we cannot fully rule out the possibility that individual psychotherapists may have participated more than once (eg, via both postal and online formats). While we consider the likelihood of intentional duplicate participation to be low, the risk was further minimized in the online survey through the storage of IP addresses and the technical prevention of repeated participation. Third, due to restricting our models to cases with complete data, sample sizes of the regression models varied, which may have reduced the power of our statistical models. In the linear regression model, the ratio of observations per predictor was 10, which corresponds to the lower bound of the commonly recommended range of at least 10 to 20 observations per predictor [33]. In the logistic regression model, the EPV ratio was 8.33, which is slightly below the recommended threshold of 10. However, simulation studies suggest that acceptable estimates may still be obtained with lower EPV [58]. Furthermore, results were largely consistent across crude and full models, suggesting that the findings may be robust. Although we could not find significant interaction effects (gender×professional type, age×therapy approach, and age×gender), our study was not powered to detect more subtle moderation. Such interactions may nevertheless play an important role, for instance, by also including constructs, such as digital affinity or practice infrastructure. Future studies with larger and more diverse samples should investigate these mechanisms more comprehensively. The fourth limitation concerns the operationalization of the prescribing of DHA-MDs. Although dichotomizing the outcome variable (“prescribing vs no prescribing”) inevitably discards information, the distribution in our sample left little variability beyond this binary categorization. Of the 271 valid responses, nearly 188 (69.4%) psychotherapists reported no prescription, and an additional 24 (8.9%) reported only 1 prescription, while higher counts were rare. As a result, attempts to fit count data models (eg, zero-inflated negative binomial regression) did not converge. Therefore, the binary operationalization provided a more robust and interpretable approach, although we could not explore potential dose-response relationships. Fifth, we used a self-designed questionnaire and assessed the perceived need for DHA-MDs with a single 11-point item that was deducted from the reach, effectiveness, adoption, implementation, and maintenance model [59,60] and refined after pilot testing. The format was chosen to allow sufficient sensitivity, consistent with numerical rating scales widely used in mental health research [31,32]. Nevertheless, the lack of formal psychometric validation remains a limitation, and future studies should develop and validate standardized multi-item instruments. Sixth, we asked psychotherapists how many patients they usually prescribe a DHA-MD to in a regular quarter instead of asking for the exact number of DHA-MDs. Thus, we were not able to consider multiple prescriptions in terms of follow-up prescriptions or several different DHA-MDs for the same patient, which might have been interesting to differentiate and thus may be a subject for future studies. Although participants were explicitly encouraged to consult their practice management system when reporting prescription frequencies, we cannot rule out nondifferential recall bias because prescribing may have been estimated rather than taken directly from practice documentation. Although we clearly defined DHA (German: DiGA) and DHA-MD (German: PsyDiGA) in the survey materials, awareness of these applications among mental health professionals is still limited, and some variability in interpretation cannot be excluded. Furthermore, other digital applications available through selective contracts (German: Selektivverträge) may also be relevant for the field of digital mental health care but were not the focus of this study.

Regarding generalizability, it should be noted that the findings of this study are specific to the German DHA-MD (German: DiGA) framework, which provides a unique legal basis for prescribing and reimbursement of DHA-MDs. Nevertheless, they may also be relevant for other countries that have recently introduced similar initiatives, such as France, Belgium, or the United Kingdom [7,9]. At the same time, cross-national differences in health care systems, reimbursement structures, and professional training limit the direct transferability of our findings. Therefore, future cross-national research is needed to examine whether the patterns observed in Germany can be replicated in other contexts.

In conclusion, we investigated the characteristics of psychotherapists and their practices as predictors of perceived need and prescribing of DHA-MDs among psychotherapists in a cross-sectional cohort in Germany. Professional group, age, psychotherapy approach, and treatment focus predicted perceived need for DHA-MDs. Psychotherapy approach, size of service mandate, practice type, and community size predicted prescribing of DHA-MDs. Our results elucidate how psychotherapists, as an important group of prescribers of DHA-MDs, evaluate their use in the real-world setting and how their evaluation differs among specific groups of psychotherapists.

## Data Availability

The datasets generated or analyzed during this study are available from the corresponding author on reasonable request.

## Appendix

Multimedia Appendix 1 Recoding map and category counts for logistic regression models.

Multimedia Appendix 2 Separate linear regression models for psychotherapists´ characteristics as predictors of perceived need for digital mental health applications (crude models).

Multimedia Appendix 3 Logistic regression models for psychotherapists’ characteristics as predictors of prescribing a digital mental health application (crude models).

## Abbreviations

DHA: digital health application
DHA-MD: digital health application for mental disorders
DiGAPS: Evaluation of Digital Health Applications for Mental Disorders (German: Digitale Gesundheitsanwendungen für psychische Erkrankungen auf dem Prüfstand)
EPV: events per variable
mHealth: mobile health
OR: odds ratio
