# Supplementary material for: Predictors of Perceived Need for and Prescribing of Digital Health Applications for Mental Disorders Among Psychotherapists in Germany: Cross-Sectional Survey Study
Source: J Med Internet Res. 2025 Nov 20;27:e78597. doi: 10.2196/78597 (PMC12679071; doi:10.2196/78597)
Supplement: Multimedia Appendix 1 [file jmir_v27i1e78597_app1.docx]

Multimedia Appendix 1: Recoding map and category counts for logistic regression models.

| Predictor variable | Original categories (*n* events / *n* total) | Recoded categories (analytic sample) |
| --- | --- | --- |
| Gender | Woman (65/187)  Man (17/77) | Unchanged |
| Professional group | Psychologist (60/201)  Medical doctor (psychiatry/psychosomatic medicine) (23/64)  Other (0/6) | ´Other´ was dropped due to 0 events |
| Age focus | Adult (74/238)  Child and adolescent (1/3)  Both (6/18) | Predictor was not included as current DHA-MD are only eligible for adults. |
| Psychotherapy approach | Psychodynamic (27/141)  Behavioral (42/98)  Systemic or other (4/17) | ´Psychodynamic´ and ´systemic or other´ were collapsed into ´Psychodynamic, systemic or other´. |
| Size of service mandate | ≤ Half (42/159)  > Half (38/88)  None (3/17) | ´None´ was collapsed into ´≤ Half´. |
| Treatment focus | Psychotherapeutic (66/243)  Psychopharmacological (8/9)  Both (9/13) | Crude models: unchanged  Full models: not included |
| Practice type | Single Practice (46/163)  Group Practice (18/35)  Medical Care Center (5/11)  Joint Practice (13/55) | ´Group Practice´ and ´Medical Care Center´ were collapsed into ´Group Practice or Medical Care Center´. |
| Community size | Rural community (<5.000 inhabitants) (9/20)  Small town (5.000 – 20.000 inhabitants) 21/52)  Medium-sized town (>20.000 – 100.000 inhabitants) (25/56)  Large city (>100.000 inhabitants) (27/136) | ´Rural community (<5.000 inhabitants)´ and ´Small town (5.000 – 20.000 inhabitants)´were collapsed into ´Rural community or small town (≤20.000 inhabitants). |
| Age | Continuous (mean, SD) | Continuous (unchanged) |

Note. In the variable `gender`, for ´nonbinary´ there was only 1 case, thus, we set it to missing and skipped the category, but kept the person in the dataset.
