# Supplementary material for: Predictors of Perceived Need for and Prescribing of Digital Health Applications for Mental Disorders Among Psychotherapists in Germany: Cross-Sectional Survey Study
Source: J Med Internet Res. 2025 Nov 20;27:e78597. doi: 10.2196/78597 (PMC12679071; doi:10.2196/78597)
Supplement: Multimedia Appendix 2 [file jmir_v27i1e78597_app2.docx]

Multimedia Appendix 2: Separate linear regression models for psychotherapists´ characteristics as predictors for perceived need of digital mental health applications (crude models).

| Predictor variable | *n* | *b* | 95% CI | | *p*-value |
| --- | --- | --- | --- | --- | --- |
| Gender | 306 |  |  |  |  |
| Woman (ref.) | 221 | – | – | – | – |
| Man | 85 | **-0.58** | [-1.11, | -0.04] | **.04** |
| Professional group | 308 |  |  |  |  |
| Psychologist (ref.) | 228 | – | – | – | – |
| Medical doctor (psychiatry/psychosomatic medicine) | 72 | 0.16 | [-0.41, | 0.73] | .58 |
| Other | 8 | -0.70 | [-2.21, | 0.81] | .36 |
| Age focus | 298 |  |  |  |  |
| Adult (ref.) | 267 | – | – | – | – |
| Child and adolescent | 11 | 0.56 | [-0.72, | 1.84] | .39 |
| Both | 20 | 0.71 | [-0.25, | 1.68] | .15 |
| Psychotherapy approach | 294 |  |  |  |  |
| Psychodynamic | 162 | **-1.84** | [-2.31, | -1.38] | **<.001** |
| Behavioral (ref.) | 115 | – | – | – | – |
| Systemic or other | 17 | **-1.20** | [-2.19, | -0.21] | **.02** |
| Size of service mandate | 305 |  |  |  |  |
| ≤ Half (ref.) | 187 | – | – | – | – |
| > Half | 99 | 0.25 | [-0.27, | 0.78] | .34 |
| None | 19 | 0.54 | [-0.48, | 1.55] | .30 |
| Treatment focus | 307 |  |  |  |  |
| Psychotherapeutic (ref.) | 283 | – | – | – | – |
| Psychopharmacological | 9 | **2.13** | [0.73, | 3.53] | **<.01** |
| Both | 15 | **1.15** | [0.06, | 2.24] | **.04** |
| Practice type | 305 |  |  |  |  |
| Single Practice (ref.) | 191 | – | – | – | – |
| Group Practice | 41 | **0.95** | [0.24, | 1.65] | **<.01** |
| Medical Care Center | 11 | **1.87** | [0.60, | 3.14] | **<.01** |
| Joint Practice | 62 | 0.14 | [-0.46, | 0.74] | .64 |
| Community size | 304 |  |  |  |  |
| Rural community (<5.000 inhabitants) | 26 | 0.62 | [-0.26, | 1.50] | .16 |
| Small town (5.000 – 20.000 inhabitants) | 63 | **0.84** | [0.22, | 1.46] | **<.01** |
| Medium-sized town (>20.000 – 100.000 inhabitants) | 69 | **0.78** | [0.18, | 1.38] | **.01** |
| Large city (>100.000 inhabitants) (ref.) | 146 | – | – | – | – |
| Age | 306 | **-0.05** | [-0.07, | -0.03] | **<.001** |
| Years of professional experience | 307 | **-0.03** | [-0.05, | -0.01] | **<.01** |

Note. CI – confidence interval. In the variable `gender`, for ´nonbinary´ there was only 1 case, thus, we set it to missing and skipped the category, but kept the person in the dataset. Due to missing data, ns vary for the separate linear regression models. Reference categories are indicated as “(ref.)”.
