# Supplementary material for: Predictors of Perceived Need for and Prescribing of Digital Health Applications for Mental Disorders Among Psychotherapists in Germany: Cross-Sectional Survey Study
Source: J Med Internet Res. 2025 Nov 20;27:e78597. doi: 10.2196/78597 (PMC12679071; doi:10.2196/78597)
Supplement: Multimedia Appendix 3 [file jmir_v27i1e78597_app3.docx]

Multimedia Appendix 3. Logistic regression models for psychotherapists’ characteristics as predictors of prescribing a digital mental health application (crude models).

| Predictor variable | Events per predictor (prescribing: 1=yes) | OR | 95% CI | | *p*-value |
| --- | --- | --- | --- | --- | --- |
| Gender | 82 |  |  |  |  |
| Woman (ref.) | 65 | – | – | – | – |
| Man | 17 | **0.53** | [0.29, | 0.99] | **.045** |
| Professional group | 83 |  |  |  |  |
| Psychologist (ref.) | 60 | – | – | – | – |
| Medical doctor (psychiatry/psychosomatic medicine) | 23 | 1.32 | [0.73, | 2.39] | .36 |
| Psychotherapy approach | 77 |  |  |  |  |
| Psychodynamic, systemic, or other | 31 | **0.28** | [0.16, | 0.48] | **<.001** |
| Behavioral (ref.) | 46 | – | – | – | – |
| Size of service mandate | 83 |  |  |  |  |
| ≤ Half (ref.) | 45 | – | – | – | – |
| > Half | 38 | **2.21** | [1.29, | 3.80] | **<.01** |
| Treatment focus | 83 |  |  |  |  |
| Psychotherapeutic (ref.) | 66 | – | – | – | – |
| Psychopharmacological | 8 | **21.45** | [2.63, | 174.85] | **<.01** |
| Both | 9 | **6.03** | [1.80, | 20.62] | **<.01** |
| Practice type | 82 |  |  |  |  |
| Single Practice (ref.) | 46 | – | – | – | – |
| Group Practice or Medical Care Center | 23 | **2.54** | [1.30, | 4.98] | **<.01** |
| Joint practice | 13 | 0.79 | [0.39, | 1.60] | .51 |
| Community size | 82 |  |  |  |  |
| Rural community or small town (≤20.000 inhabitants) | 30 | **2.88** | [1.54, | 5.41] | **<.01** |
| Medium-sized town (>20.000 – 100.000 inhabitants) | 25 | **3.26** | [1.66, | 6.39] | **<.01** |
| Large city (>100.000 inhabitants) (ref.) | 27 | – | – | – | – |
| Age | 83 | **0.97** | [0.95, | 0.99] | **<.01** |
| Years of professional experience | 83 | **0.97** | [0.95, | 1.00] | **.02** |

Note. CI – confidence interval; OR – odds ratio. In the variable `gender`, for ´nonbinary´ there was only 1 case, thus, we set it to missing and skipped the category, but kept the person in the dataset. Reference categories are indicated as “(ref.)”. Due to missing data, ns vary for the separate logistic regression models as follows: gender: n=264, professional group: n=265, age focus: n=265, psychotherapy approach: n=256, size of service mandate: n=264, treatment focus: n=265, practice type: n=264, community size: n=264, age: n=264, years of professional experience: n=265.
